# Diabetische Nierenerkrankung (Update 2026): Leitlinie in Zusammenarbeit der Österreichischen Diabetes Gesellschaft und der Österreichischen Gesellschaft für Nephrologie

**DOI:** 10.1007/s00508-025-02671-y

**Published:** 2026-04-30

**Authors:** Harald Sourij, Felix Aberer, Marlies Antlanger, Johanna Brix, Daniel Cejka, Martin Clodi, Roland Edlinger, Kathrin Eller, Sabine Horn, Susanne Kaser, Alexandra Kautzky-Willer, Michael Leutner, Rainer Oberbauer, Markus Pirklbauer, Alexander R. Rosenkranz, Sabine Schmaldienst, Harald Stingl, David Strobl, Marcus Säemann

**Affiliations:** 1 Klinische Abteilung für Endokrinologie und Diabetologie, Cardiometabolic Trials Unit, Medizinische Universität Graz, Graz, Österreich; 2 Klinische Abteilung für Endokrinologie und Diabetologie, Medizinische Universität Graz, Graz, Österreich; 3 Universitätsklinik für Innere Medizin 2, Kepler Universitätsklinikum Linz, Linz, Österreich; 4 1. Medizinische Abteilung mit Diabetologie, Endokrinologie und Nephrologie, Klinik Landstraße, Wien, Österreich; 5 Abteilung für Innere Medizin 3, Ordensklinikum Linz, Elisabethinen, Linz, Österreich; 6 Abteilung für Innere Medizin, Krankenhaus Barmherzige Brüder Linz, Linz, Österreich; 7 3. Medizinische Abteilung mit Stoffwechselerkrankungen und Nephrologie, Klinik Hietzing, Wien, Österreich; 8 Klinische Abteilung für Nephrologie, Universitätsklinik für Innere Medizin, Medizinische Universität Graz, Graz, Österreich; 9 Abteilung für Innere Medizin, LKH Villach, Villach, Österreich; 10 Universitätsklinik für Innere Medizin I, Medizinische Universität Innsbruck, Innsbruck, Österreich; 11 Klinische Abteilung für Endokrinologie und Stoffwechsel, Universitätsklinik für Innere Medizin III, Medizinische Universität Wien, Wien, Österreich; 12 Klinische Abteilung für Nephrologie und Dialyse, Universitätsklinik für Innere Medizin III, Medizinische Universität Wien, Wien, Österreich; 13 Universitätsklinik für Innere Medizin IV – Nephrologie und Hypertensiologie, Medizinische Universität Innsbruck, Innsbruck, Österreich; 14 1. Medizinische Abteilung, Klinik Favoriten, Wien, Österreich; 15 Abteilung für Innere Medizin, Landesklinikum Baden-Mödling, Baden, Österreich; 16 1. Medizinische Abteilung, Landesklinikum Stockerau, Stockerau, Österreich; 17 6. Medizinische Abteilung mit Nephrologie & Dialyse, Klinik Ottakring, Wien, Österreich

**Keywords:** Diabetes mellitus Typ 1, Diabetes mellitus Typ 2, Diabetische Nierenerkrankungen, Chronische Nierenerkrankungen, Dialyse, Type 1 diabetes, Type 2 diabetes, Diabetic kidney disease, Chronic kidney disease, Dialysis

## Abstract

Epidemiologische Untersuchungen zeigen, dass etwa 2–3 % aller Österreicher*innen einen Diabetes mellitus mit Nierenbeteiligung aufweisen. Dies betrifft somit in Österreich etwa 250.000 Menschen. Das Risiko des Auftretens und Fortschreitens der diabetischen Nierenerkrankung (DKD) kann durch Lebensstilinterventionen und Optimierung des arteriellen Blutdrucks, Blutzuckers und durch spezifische Medikamentenklassen vermindert werden. In dieser gemeinsamen Leitlinie der Österreichischen Gesellschaften für Nephrologie und Diabetologie werden die Definition, entsprechende Diagnostik und therapeutische Strategien bei DKD vorgeschlagen.

## Abstract (en)

Epidemiological investigations have shown that approximately 2–3% of all Austrians have diabetes mellitus with renal involvement (diabetic kidney disease, DKD). Therefore, this concerns approximately 250,000 people in Austria. The risk of occurrence and progression of DKD can be attenuated by lifestyle interventions as well as optimization of blood pressure, blood glucose control and specific drug classes. These guidelines represent the joint recommendations of the Austrian Diabetes Association and the Austrian Society of Nephrology for the definition, diagnostics and treatment strategies of DKD.

## Hintergrund

Die diabetische Nierenerkrankung (DKD) wird anhand einer persistierenden Erhöhung der Harn-Albuminausscheidung (Albumin-Kreatinin-Ratio ≥ 30 mg/g) und/oder einer reduzierten eGFR (errechneten glomeruläre Filtrationsrate; < 60 ml/min/1,73 m^2^) im Sinne auch der Definition einer chronischen Nierenerkrankung („chronic kidney disease“ [CKD]) bei vorliegendem Diabetes mellitus und dem Fehlen einer anderen primären Ursache für eine CKD diagnostiziert.

Diabetes mellitus und vaskulär hypertensive Erkrankungen stellen die häufigsten Ursachen terminaler Nierenfunktionseinschränkung in Österreich dar [1]. Strategien zur Verhinderung des Auftretens bzw. der Progression sind daher von größter Bedeutung. Im Jahr 2023 waren laut österreichischem Dialyse- und Transplantationsregister (OEDTR) 19,8 % der Neuzugänge zur Dialyse Menschen mit Diabetes mellitus (16,7 % Typ-2-Diabetes [T2D], 3,1 % Typ-1-Diabetes [T1D]). Es muss darauf hingewiesen werden, dass die Inzidenz der Menschen mit chronischer Dialyse und T2D seit 2007 kontinuierlich rückläufig ist, die Prävalenz jedoch weiterhin steigt oder stabil bleibt [2, 3]. Letzteres wird durch Daten aus dem Österreichischen Dialyse- und Transplantationsregister unterstrichen, die eine Steigerung des Überlebens von Menschen mit T2D im Zeitraum von 1998 auf 2007 um insgesamt ein Jahr zeigen konnten [4].

## Die Nierenerkrankung bei Menschen mit Typ-1-Diabetes (T1D)

Der Verlauf der Nierenerkrankung bei Menschen mit T1D ist weniger variabel als bei Menschen mit T2D. Bei T1D ist eine optimale/intensivierte Blutzuckereinstellung die wichtigste Maßnahme zur CKD-Prävention und in frühen Stadien der CKD auch der Intervention. Bei optimaler Einstellung (HbA_1c_ < 7 % [53 mmol/mol]) kam es in einer großen Interventionsstudie, in der nur über 6,5 Jahre ein glykämischer Unterschied aufrechterhalten wurde, selbst nach 30 Jahren noch zu einer 36- bis 76 %igen Reduktion der mikrovaskulären Komplikationen im Vergleich zur Gruppe die in den initialen Jahren ein HbA_1c_ ~ 9 % hatte [5]. Die Inzidenz der terminalen Nierenfunktionseinschränkung in der intensiv behandelten Gruppe lag bei 11/1000 Patient*innen [6]. Neben einer optimalen glykämischen Kontrolle ist auch auf eine adäquate Behandlung weiterer Risikofaktoren wie Bluthochdruck, Dyslipidämie, erhöhtes Körpergewicht und auf eine Lifestylemodifikation zu achten [7, 8]. Sobald entweder eine Hypertonie oder eine Albuminurie (ab Stadium A2, entsprechend einer Albumin-Kreatinin-Ratio von > 30 mg/g im Harn) vorliegen, gilt die medikamentöse Blockade des Renin-Angiotensin-Aldosteron-Systems (RAAS) als gesicherte Therapie zur Nephroprotektion (Reduktion der Albuminurie und Reduktion des GFR-Abfalls) [9–12].

## Die Nierenerkrankung bei Menschen mit Typ-2-Diabetes (T2D)

Die Prävalenz des T2D in Österreich ist nicht genau bekannt, liegt aber etwa bei 8 % [13]. Etwa 25 % dieser Personen haben auch eine chronische Nierenkrankheit („chronic kidney disease“ [CKD]) Stadium G3 oder höher (eGFR < 60 ml/min/1,73 m^2^) [14] und/oder eine Albuminurie, diese werden in weiterer Folge unter dem Begriff diabetische Nierenerkrankung („*diabetic kidney disease*“ [DKD]) zusammengefasst. Durch das erhöhte Mortalitätsrisiko von Menschen mit T2D wie auch das gesteigerte Mortalitätsrisiko der CKD selbst („competing risk of death“) versterben viele, bevor sie das Stadium der terminalen Nierenfunktionseinschränkung erreichen.

Die CKD bei T2D ist ätiologisch heterogener als bei Menschen mit T1D, somit sind der Verlauf und die Prognose schwieriger abzuschätzen. Aufgrund der meist schon längeren Zeitspanne zwischen Beginn der gestörten Glukosestoffwechsellage und Diagnose des T2D können zum Zeitpunkt der T2D-Diagnosestellung bereits eine CKD und/oder eine Albuminurie vorliegen. Ohne spezielle Intervention entwickeln ca. 20–40 % der Betroffenen eine Albuminurie Stadium A2 (s. unten) sowie eine größere Albuminurie bzw. Proteinurie (Stadium A3), die DKD schreitet aber insgesamt nur bei etwa 20 % dieser Patient*innen innerhalb von 20 Jahren zu einer terminalen Niereninsuffizienz fort [15]. Dies ist teilweiseauch dadurch bedingt, dass das Vorliegen einer Albuminurie *oder eGFR-Reduktion* mit einer erhöhten Inzidenz kardiovaskulärer Morbidität und Mortalität einhergeht, sodass viele Menschen schon vor dem Auftreten einer terminalen Niereninsuffizienz versterben [16]. Früher ging man von einem klassischen „Durchlaufen“ aller Stadien bis zur Entwicklung der terminalen Niereninsuffizienz aus und betonte die Wertigkeit der Albuminurie im Stadium A2 als Parameter der Frühdiagnostik. Bei vielen Menschen mit Diabetes mit eingeschränkten Nierenfunktionsparametern findet sich jedoch keine Albuminurie [15], sodass hier primär eine renale mikro-/makrovaskuläre Komponente mit extraglomerulären Schäden anzunehmen ist.

## Geschichte und Spektrum der diabetischen Nierenerkrankung

Mitte des 20. Jahrhunderts wurde der Begriff der diabetischen Nephropathie als klinisches Syndrom, basierend auf interkapillärer oder nodulärer Glomerulosklerose (Kimmelstiel-Wilson) bei Patient*innen mit längerer Diabetesdauer, persistierender Albuminurie, Hypertonie, Retinopathie und progressivem Nierenfunktionsverlust geprägt [17, 18]. Dieser wurde in den letzten Jahren durch die klassischen 5 Stadien des natürlichen Krankheitsverlaufes einer CKD ergänzt [19]. Obwohl dieses Modell und der Krankheitsverlauf primär auf Daten von Menschen mit T1D basierte [20, 21], wurde es auch bei Menschen mit T2D verwendet [22]. Mittlerweile ist aber klar, dass mehr als 50 % der Menschen mit T2D in Langzeitbeobachtungen eine GFR < 60 ml/min/1,73 m^2^ ohne vorangehende Albuminurie entwickeln [23–26] bzw. der Verlauf der Albuminurie nicht immer mit dem Nierenfunktionsverlust korreliert [27]. Ähnliche Beobachtungen gibt es auch für T1D [28]. Histopathologische Untersuchungen bei Menschen mit T2D und CKD weisen auf ein vielfältiges Spektrum an Nierenerkrankungen hin. Diese erwähnte Heterogenität renaler Pathologien bei Patient*innen mit Diabetes mellitus hat vor allem dazu geführt den Begriff diabetische Nephropathie zu verlassen, da damit eine homogene klinisch-pathologische Entität suggeriert wurde, und den Begriff der diabetischen Nierenerkrankung zu verwenden. Das Ausmaß der Albuminurie weist die engste Korrelation mit dem Auftreten einer histopathologischen DKD auf [29]. Von der *Renal Pathology Society* wurde zwar eine Klassifikation auf Basis von Biopsien von Menschen mit T1D und T2D erstellt [30], allerdings wird diese Klassifikation in der klinischen Routinebefundung im Allgemeinen wie auch in Österreich speziell nicht verwendet.

**Abb. 1:**
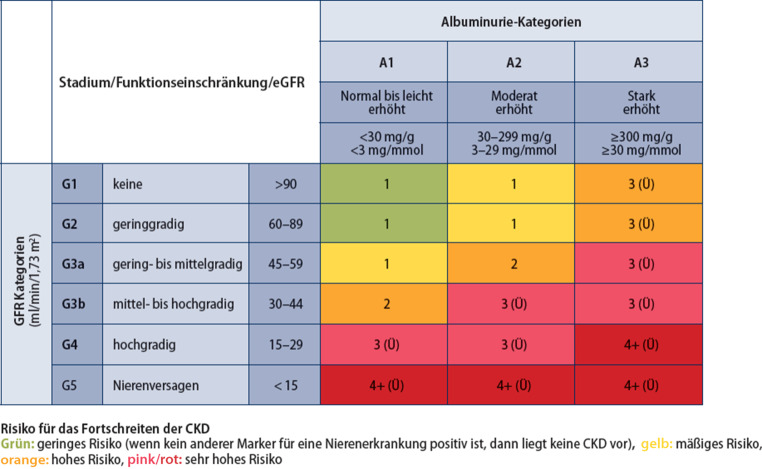
Stadien der CKD und Risiko für Progression. (Adaptiert nach ADA/KIDIGO [31])

Die Stadieneinteilung der DKD entspricht der klassischen Einteilung der CKD-Stadien nach KDIGO: die geschätzte glomeruläre Filtrationsrate (eGFR) nach CKD-EPI (2009) wird in die Stadien G1 bis G5 (Abb. 1) eingeteilt, Stadium G3 in G3a (eGFR 45–59 ml/min/1,73 m^2^) und G3b (eGFR 30–44 ml/min/1,73 m^2^) unterteilt, da ab Stadium G3b die Morbidität und Mortalität deutlich zu steigen beginnen. Zusätzlich wird die Albuminausscheidung im Spontanharn (Albumin/Kreatinin-Ratio; UACR) in A1 (< 30 mg/g Kreatinin), A2 (30–299 mg/g) und A3 (≥ 300 mg/g) quantifiziert.

Da die Albuminurie ein kontinuierlicher renaler und v. a. kardiovaskulärer Risikomarker ist, sollten die älteren Termini der „Mikro- und Makroalbuminurie“ nicht mehr verwendet werden. Zudem gibt die Klassifizierung auch farblich das Risiko für das Auftreten kardiovaskulärer Ereignisse und das Fortschreiten der Funktionseinschränkung der Nieren bis hin zum terminalen Nierenversagen an und bietet Entscheidungshilfen zur Behandlungsindikation sowie zur Zuweisungsindikation zum/r Nephrolog*in (Abb. 1). Zudem hat sich KDIGO dafür eingesetzt, dass das individuelle Patient*innen-Risiko evaluiert werden sollte. Die sog. KFRE-Formel (Kidney Failure Risk Equation) dient als validiertes Prognoseinstrument zur Abschätzung der Wahrscheinlichkeit, innerhalb von 2 oder 5 Jahren das CKD-Stadium G5 zu erreichen, wenn die GFR unter 60 ml/min liegt. Liegt das berechnete Risiko über 3–5 %, sollte eine nephrologische Mitbeurteilung in Erwägung gezogen werden. Die Berechnung kann schnell und unkompliziert über Smartphone oder Online-Tools erfolgen – lediglich die Parameter Alter, Geschlecht, eGFR und UACR sind erforderlich. Für eGFR-Werte über 60 ml/min/1,73 m^2^ liegen weitere validierte Risikokalkulatoren vor, die auch in der entsprechenden Smartphone-App benutzt werden können (www.nieren.app oder https://kidneyfailurerisk.com/).

## Bestimmung der Nierenfunktion

Zur Beurteilung des Ausmaßes der Nierenfunktionseinschränkung sollte eine der derzeit gängigen Schätzformeln verwendet werden, welche bereits in den meisten Labors implementiert sind. Eine ausschließliche Serumkreatininbestimmung ist v. a. bei älteren Menschen oft irreführend, da keine gute Korrelation zur tatsächlichen Nierenfunktion besteht bzw. die Schwelle zu einer definierten Nierenerkrankung vermutlich niedriger anzusetzen ist als bei jungen Menschen (45 vs. 60 ml/min/1,73 m^2^). Die Basis der Berechnung soll eine nach IDMS(„isotope dilution mass spectrometry“)-Goldstandard kalibrierte Serumkreatininbestimmung sein ([32]; Tab. 1). Aktuell empfehlen die meisten Gesellschaften die CKD-EPI(Chronic Kidney Disease Epidemiology Collaboration)-Formel aus 2009 als Standard ([33, 34]; Tab. 1). Für diese Formel wurde mehrfach gezeigt, dass sie v. a. im CKD-Stadium G2–3 akkurater als die MDRD-Formel und somit besser zur Risikostratifizierung geeignet ist [35, 36].

**Tab. 1:** Darstellung der beiden am häufigsten verwendeten Schätzformeln zur Bestimmung der Nierenfunktion. (Nach [34, 37])

Glomeruläre Filtrationsrate berechnet (eGFR) – MDRD4-Formel
GFR (ml/min / 1,73 m^2^ KÖF) = 186 × (s_Cr_)^−1,154^ × Alter ^− 0,203^ × (0,724 bei Frauen)
Glomeruläre Filtrationsrate berechnet (eGFR) – CKD-EPI-Formel
GFR = 142 × min (s_cr_/κ, 1)^α^ × max (s_cr_/κ, 1)^−1,20^ × 0,9938^Alter^ × 1,012 (Frauen)

Frauen: κ = 0,7; α = −0,329, Männer: κ = 0,9; α = −0,411

*KÖF* Körperoberfläche, *s*_*Cr*_ Serumkreatinin

Da die eGFR durch die Verwendung von Kreatinin einer Vielzahl an möglichen Störfaktoren unterliegt (Muskelmasse, Ernährung, renal nichtneutrales Handling von Kreatinin, Medikamente etc.) schlägt u. a. KDIGO vor, das sensitivere Cystatin C zu verwenden und idealerweise die eGFR als Kombination von Kreatinin und Cystatin C zu errechnen (GFRcys-krea) [38]. Cystatin C hat den Vorteil von allen Zellen des Körpers produziert und nicht tubulär reabsorbiert/sezerniert zu werden. Zudem ist es nicht von der Muskelmasse des/der Patient*in abhängig. Aufgrund des noch zu hohen Preises der Cystatin C-Testung und der nicht ubiquitären Verfügbarkeit und Erfahrung schlagen wir vor, immer Cystatin C zu bestimmen, wenn prinzipielle Zweifel bzw. Implausibilität an der Kreatininmessung angebracht sind sowie bei extremen Ernährungsformen und/oder deutlichen Änderungen der Muskelmasse. Zur besseren allgemeinen Verständlichkeit schlagen die Gesellschaften auch vor, gegenüber Betroffenen die Nierenfunktion zur besseren Verständlichkeit als % Nierenfunktion zu interpretieren, was bei einem annähernden Normalwert von etwa 100 ml/min/1,73 m^2^ (90–120 ml/min/1,73 m^2^) durchaus gerechtfertigt erscheint.

## Diagnostik der diabetischen Nierenerkrankung

### Screening auf diabetische Nierenerkrankung

Bei T1D sollte das jährliche Screening auf Albuminurie 5 Jahre nach Diagnosestellung, bei T2D bereits mit der Diagnosestellung beginnen. Generell wird empfohlen, als Screening nur die Messung der Albumin/Kreatinin-Ratio (UACR) aus dem Spontanharn durchzuführen [39]. Wir empfehlen, unabhängig von der Bestimmung der Albuminurie auch eine jährliche eGFR-Bestimmung insbesondere bei T1D nach zumindest 5 Jahren Krankheitsdauer und bei allen Menschen mit T2D schon ab Diagnosebeginn durchzuführen ([40, 41]; Abb. 1).

Aufgrund der hohen individuellen Variabilität der Albuminexkretion im Harn wird zur Diagnostik der Albuminurie folgendes Vorgehen empfohlen, dabei gilt die „2 aus 3 Regel“: Wenn 2 hintereinander analysierte Urinproben innerhalb von 3 bis 6 Monaten übereinstimmend positiv (UACR ≥ 30 mg/g Kreatinin) oder negativ sind, ist eine Albuminurie nachgewiesen bzw. ausgeschlossen. Wenn die Albuminausscheidung einer Urinprobe negativ und die andere positiv ist, sollte eine dritte Urinprobe auf Albuminurie nach etwa 4 Wochen getestet werden. Zu beachten ist, dass positive Befunde z. B. auch bei akut fieberhaften Erkrankungen, Harnwegsinfekten und arterieller Hypertonie, bei Herzinsuffizienz, Menstruation und nach körperlicher Anstrengung unabhängig von möglichen diabetischen Nierenschäden möglich sind. Nachdem die Sammlung von 24-h-Harn aufwendig ist und nur wenig Zusatznutzen im Vergleich zur Bestimmung der Albumin-Kreatinin-Ratio aus dem Spontanharn, idealerweise dem Morgenharn, aufweist, hat sich letztere Methode durchgesetzt.

Es zeigt sich, dass die UACR im Verlauf deutlich schwanken kann [42]. Da aber der Verlauf so wie auch die Veränderung der eGFR über die Zeit prognostische Wertigkeit hat [43], sollten mindestens jährliche UACR-Kontrollen durchgeführt werden.

## Differenzialdiagnosen bei Menschen mit diabetischer Nierenerkrankung

Bei Menschen mit Diabetes mellitus sollte immer auch an eine mögliche andere, nichtdiabetische Ursache der Albuminurie und/oder Nierenfunktionseinschränkung gedacht werden, insbesondere wenn mindestens eines der folgenden Kriterien erfüllt ist:

- Diabetesdauer unter 5 Jahren bei T1D,
- fehlende (insbesondere proliferative) oder nur milde diabetische Retinopathie vor allem bei Menschen mit T1D,
- pathologisches Harnsediment mit Mikro- oder Makrohämaturie (insbesondere Akanthozytennachweis und Erythrozytenzylinder),
- dauerhaft gute Blutzuckerkontrolle,
- sehr rasche Zunahme der Albuminurie, definiert als Klassenwechsel der Albuminurie (A1 auf A2 oder A3 sowie A2 auf A3 innerhalb kurzer Zeit),
- rascher Abfall der eGFR (≥ 5 ml/min/1,73 m^2^/Jahr),
- Auffälligkeiten in der Nierensonographie, welche an eine andere Nierenpathologie denken lassen.

Differenzialdiagnostisch häufig zu erwägende Nierenerkrankungen, die statt oder auch zusätzlich zu einer DKD bestehen können, sind eine hypertensive oder eine ischämische Nephropathie als Folge einer atherosklerotische Veränderung der Nierengefäße. Bei ausgeprägter Albuminurie und/oder Mikrohämaturie ist differenzialdiagnostisch an andere renale Erkrankungen zu denken (u. a. Vaskulitis, Glomerulonephritis, Amyloidose), die einer gezielten und oft auch raschen Therapie bedürfen, weshalb hier eine weitere zügige nephrologische Abklärung notwendig ist. Eine Indikation zur Nierenbiopsie als diagnostischer Goldstandard sollte in Anbetracht der Vielfalt anderer ernsthafter Nierenerkrankungen in diesen Fällen großzügig gestellt werden.

## Therapeutische Gesichtspunkte bei Menschen mit diabetischer Nierenerkrankung

### Ernährung

Hinsichtlich der Eiweißzufuhr mit der Nahrung werden nach den KDIGO- und den ADA-Leitlinien 0,8 g/kg Körpergewicht sowie die Vermeidung der Überschreitung von 1,3 g/kg Körpergewicht empfohlen [8, 44, 45]. Eine noch niedrigere Eiweißzufuhr scheint keinen weiteren Nutzen zu haben [46]. Zusätzlich wird ein Meiden von industriell verarbeiteten Lebensmitteln [47] empfohlen sowie eine Reduktion der Kochsalzzufuhr auf 5 g/Tag vorgeschlagen [44]. Es sollte dazu angemerkt werden, dass auch Studien bei Menschen mit T1D und mit T2D die Kochsalzrestriktion kritisch hinterfragen, und die Studienlage in dieser Population dafür nur geringe Evidenz liefert [44, 48, 49]. Weitere Studien zum therapeutischen Nutzen einer Kochsalzrestriktion sind daher notwendig, bevor solide Empfehlungen gemacht werden können.

Verschiedene Diäten werden diskutiert, um das kardiovaskuläre Risiko von Menschen mit Diabetes mellitus zu senken. Diäten sind naturgemäß schwierig zu standardisieren, und die Studienlage ist sehr heterogen. Eine allgemeingültige Diätempfehlung, die für alle Patient*innen gut passend und auch praktisch umsetzbar ist, kann daher aktuell nicht gegeben werden [50–53].

Erwiesenermaßen trägt Gewichtsreduktion bei Adipositas ab Grad II (BMI > 35 kg/m^2^) durch ein bariatrisch chirurgisches Vorgehen [54] zu einer Verbesserung der Stoffwechsellage oder sogar zur Diabetesremission und zu einer deutlichen Reduktion der Risken für diabetische Komorbiditäten bei [55].

### Kardiovaskuläres Risiko

Die Diagnose einer CKD bei bestehendem Diabetes mellitus steigert substanziell Morbidität und Mortalität [16]. Ein Großteil der erhöhten Mortalität ist auf kardiovaskuläre Erkrankungen zurückzuführen, obwohl die nichtkardiovaskuläre Mortalität ebenso erhöht ist. Albuminurie und eGFR sind unabhängige und zusätzliche Risikofaktoren für kardiovaskuläre Ereignisse, kardiovaskuläre Mortalität und Gesamtmortalität [16, 56]. Dies führt zur Empfehlung, dass Menschen mit Diabetes mellitus und CKD wie kardiovaskuläre Sekundärpräventionspatient*innen behandelt werden sollten [57]. Die Mechanismen, durch die eine DKD das kardiovaskuläre Risiko beeinflusst, umfassen traditionelle Risikofaktoren (Hyperglykämie, Hypervolämie und Hypertonie, Lipoproteinmetabolismus, systemische Inflammation, oxidativer Stress und endotheliale Dysfunktion, Rauchen, Umweltfaktoren) wie auch Mechanismen spezifisch im Zusammenhang mit der Nierenfunktionseinschränkung (z. B. Urämietoxine, Anämie und Störungen des Knochen- und Mineralstoffwechsels) [58]. Diese Überlegungen fließen in die unten angeführten Therapieempfehlungen ein.

### Lipidstoffwechsel

Die DKD wird durch Störungen des Lipidmetabolismus in Zusammenhang mit einer Abnahme der Nierenfunktion abhängig vom Stadium der CKD begleitet. Wie bereits erwähnt, führt das Vorliegen einer CKD auch zu einem deutlichen Anstieg des kardiovaskulären Risikos [56]. LDL-Cholesterin (LDL-C) ist ein etablierter Risikofaktor für kardiovaskuläre Erkrankungen in der Allgemeinbevölkerung. Bei diabetischer Nierenerkrankung wird eine quantitative Verschiebung im Lipidprofil zu erhöhten Triglyzeriden, niedrigem HDL-Cholesterin inklusive qualitativen Änderungen des HDL-Cholesterins sowie erhöhten Spiegeln an oxidiertem LDL-Cholesterin gefunden [59].

Das Ausmaß der LDL-C-Senkung bei der CKD-Population mit Statinen ist vergleichbar mit Personen mit erhaltener Nierenfunktion [60]. Klinische Untersuchungen und entsprechende Metaanalysen bei nichtdialysepflichtiger CKD zeigen, dass kardiovaskuläre Ereignisse und/oder Mortalität durch Statine bzw. die Kombination Statine/Ezetimib im Vergleich zu Placebo gesenkt werden [60–62]. Der günstige Effekt scheint nicht durch den Diabetes modifiziert zu sein. Während der kardiovaskuläre Benefit durch Statine bei CKD gut dokumentiert ist, haben Statine keine progressionsverzögernde Wirkung hinsichtlich der Nierenfunktion [63]. Statine werden daher bei allen Menschen mit Diabetes und nichtdialysepflichtiger CKD empfohlen [64, 65]. Da jedoch der kardiovaskuläre Nutzen einer Statintherapie mit sinkender eGFR abnimmt, sollte eine Statintherapie in möglichst frühen Stadien der CKD begonnen werden.

Rezente Daten haben auch gezeigt, dass PCSK9-Hemmer bei Personen im CKD-Stadium G3–5 (mit und ohne T2D) vergleichbare lipidsenkende Wirkungen und Sicherheitsprofile wie bei Patient*innen mit einer eGFR > 60 ml/min/1,73 m^2^ aufweisen [66–68], und sind daher für die Therapie in diesem Kollektiv auch zugelassen. Die Small-interfering-RNA-Therapie mit Inclisiran zeigt ebenfalls bei CKD vergleichbare Sicherheits- und Effektivitätsdaten wie bei Menschen ohne CKD, auch wenn für diese Substanz noch die Endpunktdaten fehlen [69]. Zudem scheint die Effektivität von Inclisiran bei höhergradiger Proteinurie reduziert zu sein, weshalb für diese Patient*innen die beiden zugelassenen PCSK9-Inhibitoren vorteilhaft sind. Die kardiovaskulären Vorteile einer Lipidtherapie mit Bempedoinsäure wurden in der CLEAR-OUTCOME-Studie untersucht. Etwa 21 % der Studienteilnehmer*innen hatten eine CKD im Stadium 3, der kardiovaskuläre Benefit war in dieser Gruppe nicht unterschiedlich zu jenen ohne CKD [70]. Während Bempedoinsäure zu geringen Anstiegen von Serumkreatinin durch vermutlich Hemmung der tubulären Kreatininsekretion führen kann, scheinen diese zu keinen klinisch relevanten Nierenfunktionsstörungen zu führen [71].

Bei Dialysepflichtigkeit haben 3 große klinische Studien (4D, Aurora und SHARP) keinen Vorteil einer Statintherapie auf den kombinierten kardiovaskulären Endpunkt (3-Punkt-MACE) zeigen können [72, 73]. Im Kollektiv von Menschen mit Diabetes und Dialysepflicht zeigt sich jedenfalls keine Indikation für die Neueinleitung einer Statintherapie, wobei eine bereits etablierte Therapie insbesondere bei kardiovaskulärer Erkrankung fortgeführt werden kann.

### Betreuung von Patient*innen mit DKD

Eine nephrologische Begutachtung ist bei Unklarheit über die Ätiologie der Nierenerkrankung und/oder rascher CKD-Progression indiziert. Prinzipiell sollte auch unabhängig vom GFR-Stadium bei einer Albuminurie im Stadium A3 sowie bei CKD G3b mit A2/A3 und G4 unabhängig von der Albuminurie eine nephrologische Vorstellung bzw. Betreuung erfolgen, ebenso bei einem 5-Jahres-Risiko von > 3–5 % nach KFRE. Ab CKD-Stadium G3 sollte eine gemeinsame Betreuung durch Diabetolog*innen und Nephrolog*innen erwogen werden und zusätzlich Augenmerk auf mögliche renale Folgeerkrankungen gelegt werden (z. B. renale Anämie, CKD-mineral-bone-disorder-Syndrom etc.).

Ab CKD-Stadium G4 (bzw. einer GFR < 20 ml/min/1,73 m^2^) ist auch die Eignung für eine alleinige Nierentransplantation oder eine kombinierte Nieren- und Pankreastransplantation (bevorzugt bei T1D, aber auch in ausgewählten Fällen bei T2D möglich [74]) zu prüfen. Gleiches gilt für das 2-Jahres-Risiko von > 10 % nach KFRE. Optimal ist durchwegs auch eine präemptive Transplantation (Lebend- oder Post-mortem-Spende). Insbesondere bei Menschen mit T1D ist aufgrund des exzessiven kardiovaskulären Risikos eine rechtzeitige Evaluation für eine Transplantation anzustreben, um die Zeit an der Hämo- oder Peritonealdialyse so kurz wie möglich zu halten oder diese idealerweise überhaupt zu vermeiden.

## Antihyperglykämische Therapie

Bei Menschen mit T1D oder T2D sollte möglichst eine optimale Stoffwechselsituation angestrebt werden [75]. In der Primärprävention sind niedrigere HbA_1c_-Werte zu fordern als in fortgeschrittenen Stadien der CKD und in der Sekundärprävention. Hier hat sich in den Studien ein HbA_1c_-„Zielkorridor“ von 6,5–7,5 % als sinnvoll erwiesen. Unabhängig davon sollte aufgrund der Vorgeschichte, Komorbiditäten, Hypoglykämieneigung und diabetischer Folgeerkrankungen (Retinopathie, Neuropathie) insbesondere bei älteren Menschen eine individualisierte Festlegung der Therapieziele erfolgen. Bei nachlassender Nierenfunktion ist besonders das erhöhte Risiko der Hypoglykämie zu berücksichtigen. Die Wahl antidiabetischer und anderer Medikamente bedarf bei eingeschränkter Nierenfunktion erhöhter Aufmerksamkeit, da Zulassungseinschränkungen und Kontraindikationen vorliegen können.

### Renoprotektive antihyperglykämische Substanzen

**Abb. 2:**
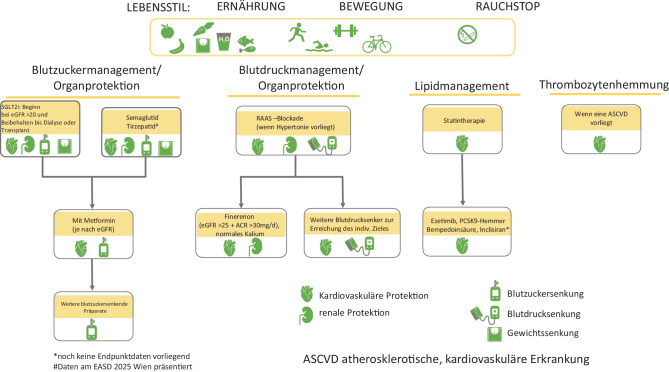
Zusammenfassung der Behandlungsempfehlungen

Einige antihyperglykämische Substanzen haben direkte renale Effekte gezeigt, die sich nicht alleine durch die Blutzuckersenkung erklären lassen [76] (Abb. 2).

### SGLT-2-Inhibitoren

Insbesondere bei Menschen mit T2D zeigten Endpunktstudien in den letzten Jahren durchschlagskräftige Daten auch im Hinblick auf die renale Risikomodifikation: Empagliflozin, ein Vertreter der SGLT2-Inhibitoren, zeigte in der EMPA-REG-OUTCOME-Studie eine signifikante, 39 %ige relative Risikoreduktion im kombinierten Endpunkt „Verschlechterung der Nephropathie“, bestehend aus Progression zu Makroalbuminurie, Verdopplung des Serumkreatinins, Beginn einer Nierenersatztherapie oder Tod renaler Ursache [77]. Canagliflozin reduzierte in der CANVAS-Studie das Risiko für einen 40 %igen eGFR-Abfall, den Beginn einer Nierenersatztherapie oder renalen Tod um relative 40 % [78]. Der nahezu idente Endpunkt (40 %iger eGFR-Abfall unter 60 ml/min/1,73 m^2^, Beginn einer Nierenersatztherapie und renaler Tod) wurde in der DECLARE-TIMI58-Studie mit Dapagliflozin um 47 % reduziert [79].

Nachdem obige Studien in Kollektiven von Menschen mit T2D mit oder ohne CKD durchgeführt wurden, untersuchten DAPA-CKD, CREDENCE und EMPA-KIDNEY die Effekte von Dapagliflozin, Canagliflozin und Empagliflozin bei Menschen mit CKD mit oder ohne T2D. In diesen Kollektiven konnte jeweils der primäre renale Endpunkt signifikant reduziert werden [80–82]. Während in der CREDENCE-Studie nur Personen mit T2D eingeschlossen wurden, hatten in DAPA-CKD 67,5 % und in EMPA-KIDNEY 46 % einen T2D.

Auch wenn die antihyperglykämischen Effekte bei SGLT-2-Inhibitoren mit abnehmender eGFR nachlassen, so bleiben die kardiorenoprotektiven Effekte bis zu einer eGFR von zumindest 20 ml/min/1,73 m^2^ erhalten, KDIGO empfiehlt sogar, die Therapie bis zur Dialysepflichtigkeit zu belassen [83].

Während SGLT-2-Inhibitoren in der Therapie von Menschen mit T2D und CKD etabliert sind, ist die Datenlage bei Menschen mit T1D geringer. In den EASE- (Empagliflozin) und DEPICT- (Dapagliflozin) Studienprogrammen konnte aufgezeigt werden, dass eine Therapie mit SGLT-2i bei Menschen mit T1D zu einer Reduktion der HbA_1c_-Konzentration, des Körpergewichts und der täglichen Insulinmenge führt, jedoch zeigte sich ein erhöhtes DKA-Risiko vor allem unter höheren Medikamentendosierungen und unter Pumpenanwender*innen, sodass aktuell keines der Präparate für die Behandlung von T1D zugelassen ist [84–86]. Gepoolte Analysen der Studien haben eine Reduktion der UACR von ≥ 30 % bei Menschen mit T1D und einer Baseline-UACR von ≥ 30 mg/g gezeigt [87, 88]. Die Ergebnisse der laufenden STENO1-Studie könnten mehr Einblick in die Sicherheit und Wirksamkeit von SGLT-Inhibition bei T1D und CKD geben (The Steno 1 study. EUCT number: 2023-505794-32). Die 22-wöchige, prospektive, kontrollierte ATTEMPT-Studie hat die Nierenfunktion und die glykämische Kontrolle bei Jugendlichen und jungen Erwachsenen mit T1D untersucht – eine Therapie mit Dapagliflozin führte zu der vorbekannten initialen Reduktion der eGFR um 8,8 ml/min/1,73 m^2^ sowie zu einer besseren glykämischen Kontrolle, verglichen mit der placebokontrollierten Gruppe. Unter der kombinierten Durchführung von Ketonkörpermessungen und Strategien zur Risikominderung gab es keinen signifikanten Unterschied beim Auftreten einer diabetischen Ketoazidose (DKA) zwischen den untersuchten Gruppen [89].

Auch wenn es sich um eine Off-label-Therapie handelt, kann der Einsatz von SGLT-2-Hemmern bei Menschen mit T1D und CKD nach sorgfältiger Aufklärung über das erhöhte DKA-Risiko in ausgewählten Fällen sinnvoll sein.

Folgende Risikominderungsstrategien müssen vor dem Einsatz von SGLT-2-Inhibitoren bei Menschen mit T1D berücksichtigt werden:

- Risikominderungsstrategien:
  - ausführliche Schulung/Ausbildung der Menschen mit T1D und der verschreibenden Kliniker*innen über DKA-Risikofaktoren, Ketonkörpermonitoring und Ketosemanagementstrategien,
  - regelmäßiges Glukose- und Ketonkörpermonitoring,
  - Risikoabwägung bei stärkeren Dosisreduktionen von Insulin,
  - Anwendung niedrigeren Dosierungen von SGLT-2-Inhibitoren,
  - Informationen über die Einhaltung „Sick days rule“ (Pausieren der Therapie bei jedweden akuten Erkrankungen).

Unter Berücksichtigung der erläuterten Risikominderungsstrategien kann unter Bedacht und besonderer Vorsicht bei nachfolgenden Patient*innen, aber auch bei weiteren Einzelfallentscheidungen eine Therapie mit SGLT-2i in Erwägung gezogen werden:

- Menschen mit T1D und CKD, bei denen es unter einer RAASi zu einer Progression der CKD kommt (Zunahme der UACR und/oder Reduktion der eGFR),
- Menschen mit T1D, CKD und/oder einer Herzinsuffizienz.

### GLP-1-Rezeptoragonisten (GLP-1-RA)

In der FLOW-Studie wurde erstmals ein GLP-1-RA, in diesem Fall Semaglutid (bis 1 mg/Woche), bei Menschen mit T2D und CKD untersucht [90]. Der primäre renale Endpunkt (eGFR-Reduktion > 50 % über zumindest 28 Tage, Dialysepflicht bzw. Nierentransplantation, eGFR < 15 ml/min/1,73 m^2^ über zumindest 28 Tage) sowie renaler bzw. kardiovaskulärer Tod konnten gegenüber Placebo um relative 24 % gesenkt werden. FLOW kann damit als Bestätigung vorheriger Studien zu Liraglutid (LEADER, präspezifizierter renaler Endpunkt [91]), Dulaglutid (REWIND, sekundärer renaler Endpunkt [92, 93]) und Semaglutid (SUSTAIN‑6, sekundärer renaler Endpunkt [94]) gesehen werden, die jedoch in Kollektiven mit T2D mit oder ohne CKD durchgeführt wurden. Gepoolte Post-hoc-Daten des SURPASS-Studienprogramms (SURPASS‑1 bis -5) mit dem GLP-1-RA/GIP-Agonisten Tirzepatid und die präsentierte SURPASS-CVOT-Studie (präsentiert EASD Wien 2025) zeigen ebenfalls vielversprechende Daten zu renalen Endpunkten [95]. Die laufende STENO1-Studie wird mehr Einblick in den Effekt eines multifaktoriellen Managements mit unter anderem GLP-1-RA auf renale und kardiovaskuläre Endpunkte bei Menschen mit T1D geben (The Steno 1 study. EUCT number: 2023-505794-32).

### DPP-4-Hemmer

DPP-4-Hemmer haben im Vergleich zu Placebo in Endpunktstudien keinen Hinweis auf einen renalen Benefit gezeigt und gelten dahingehend nicht als Therapie der Wahl zur Kardio/Nephro-Protektion.

### Therapiebesonderheiten bei nachlassender Nierenfunktion

- Orale antihyperglykämische Therapie:
  Die Auswahl von oralen Antidiabetika hat in den letzten Jahren deutlich zugenommen. Dennoch gestaltet sich die orale antidiabetische Therapie bei fortgeschrittener Nierenfunktionseinschränkung schwieriger [96]. Ebenso ist auf die erhöhte Hypoglykämieneigung bei höheren CKD-Stadien Aufmerksamkeit zu legen [97]. Im Folgenden werden die wesentlichen Substanzen bzw. Substanzgruppen aufgelistet:
- Metformin galt lange Zeit aufgrund seiner Plasmaeliminationshalbwertszeit von 4,0–8,7 h [98] bei kompletter renaler Elimination bei mittel- bis höhergradiger CKD und damit assoziierter Laktatazidosegefahr als kontraindiziert. Dies änderte sich allerdings über die letzten Jahre, da die Evidenz aus der klinischen Praxis fehlte [99, 100]. Metformin ist bei einer eGFR < 30 ml/min/1,73 m^2^ kontraindiziert, unter einer eGFR von 45 ml/min/1,73 m^2^ sollte Metformin nicht neu begonnen werden, die Dosis bei bestehender Therapie auf 1000 mg pro Tag, aufgeteilt auf zweimal 500 mg, beschränkt und die eGFR engmaschiger überwacht werden [101, 102].
- SGLT-2-Inhibitoren (Indikation Blutzuckersenkung): Nachdem wie bereits erwähnt die blutzuckersenkende Wirkung dieser Substanzklasse mit eGFR-Verringerung abnimmt, sollte ab einer eGFR von < 45 ml/min/1,73 m^2^ wenig Erwartung in eine weitere blutzuckersenkende Potenz gesetzt werden. Eine SGLT-2-Inhibitor-Therapie sollte jedoch bei einer eGFR < 45 ml/min/1,73 m^2^ fortgeführt werden (bis zur Dialysepflichtigkeit), da die günstigen reno- und kardioprotektiven Effekte jedenfalls bis zu einer eGFR von 20 ml/min/1,73 m^2^ weiterhin erhalten bleiben. Eine Therapieeinleitung erscheint bei einer eGFR von > 20 ml/min/1,73 m^2^ sinnvoll, und die Fortführung einer bereits bestehenden Therapie ist bis zum Beginn einer Nierenersatztherapie möglich [45]. Jüngere Empfehlungen legen nahe, eine SGLT2-Hemmer-Therapie bei GFR-Abfall < 20 ml/min/1,73 m^2^ fortzuführen.
- Für DPP-4-Hemmer gilt: Linagliptin kann in allen Stadien ohne Dosisanpassung gegeben werden, da es primär hepatobiliär ausgeschieden wird. Bei anderen DPP-4-Hemmern wie Sitagliptin, Vildagliptin, Saxagliptin und Alogliptin sind ab Stadium G3 Dosisanpassungen erforderlich.
- Sulfonylharnstoffe (SH) stellen aufgrund des Hypoglykämierisikos nicht das optimale orale Antidiabetikum bei Menschen mit CKD dar. Zwischen den einzelnen Substanzen gibt es erhebliche Unterschiede. Gliclazid sollte bei CKD in niedriger Dosierung begonnen und alle 4 Wochen dosistitriert werden. Glimepirid kann im Stadium CKD G1–3 in normaler Dosis, im Stadium G4 reduziert (1 mg/Tag) verabreicht werden und sollte im Stadium G5 vermieden werden [103]. Das Hypoglykämierisiko erscheint am niedrigsten bei Gliclazid [104], gefolgt von Glipizid und Glimepirid [105]. Dennoch ist insgesamt das Hypoglykämierisiko unter SH ca. 10-fach so hoch wie unter Metformin und 4‑ bis 5‑fach höher als unter Pioglitazon [106–109].
  Es sollte auf die Gabe des vorwiegend renal eliminierten Glibenclamid aufgrund der Kumulationsgefahr und dem Risiko für schwere und protrahierte Hypoglykämien verzichtet werden (heutzutage kaum mehr verwendet).
- Bei Verwendung von Repaglinid kann bis CKD-Stadium G4 ohne Dosisreduktion vorgegangen werden. Für Repaglinid gibt es im Stadium CKD G5 keine Daten.
- Pioglitazon als einzig verbleibender Vertreter der Thiazolidindione muss nicht dosisreduziert werden [103]. Pioglitazon kann entsprechend der Fachinformation bei einer Kreatinin-Clearance > 4 ml/min eingesetzt werden. Aufgrund des erhöhten Risikos für Herzinsuffizienz durch Volumenretention und dem erhöhten Risiko für periphere Frakturen bei Frauen, ist der Einsatz von Pioglitazon jedoch limitiert.
- GLP-1-Rezeptoragonisten:
  Für GLP-1-Rezeptoragonisten gilt: Dulaglutid, Liraglutid und Semaglutid können bis zu einer eGFR von 15 ml/min/1,73 m^2^ ohne Dosisanpassung angewendet werden. Für die terminale Niereninsuffizienz liegen Daten für Semaglutid aus einem kleinen Kollektiv bei Menschen mit Adipositas ohne Diabetes vor, in dem die Sicherheit der Substanz gezeigt werden konnte [110].
- GLP-1/GIP-Rezeptoragonisten:
  Der erste zugelassene GLP-1/GIP-Rezeptoragonist Tirzepatid ist auch bei Menschen mit terminaler Niereninsuffizienz entsprechend der Fachinformation ohne Dosisreduktion zugelassen.
- Insulin:
  Bei Insulinen ist auf eine mögliche Dosisreduktion in Abhängigkeit von der Nierenfunktionseinschränkung zu achten, da Insulin teilweise renal abgebaut wird und das Hypoglykämierisiko bei weit fortgeschrittener CKD generell signifikant erhöht ist.

## Blutdruckeinstellung

Eine antihypertensive Behandlung von Menschen mit Diabetes mellitus zielt darauf ab, das Auftreten und die Progression einer DKD sowie makrovaskuläre Komplikationen und vorzeitigen kardiovaskulären Tod zu vermeiden. Daraus ergeben sich folgende Therapieziele:

- Rückbildung bzw. Stabilisierung einer Albuminurie,
- Erhalt bzw. Minimierung des GFR-Abfalls und damit Verhinderung der terminalen Niereninsuffizienz,
- Reduktion kardiovaskulärer Morbidität und Mortalität. Blutdrucktherapieempfehlungen für Kinder werden im pädiatrischen Kapitel erläutert.

Der Zielblutdruck bei DKD wird mit < 140/90 mm Hg angegeben, um die kardiovaskuläre Mortalität und die Progression der CKD zu reduzieren. Zusätzlich wird von KDIGO bei einer Albuminurie ≥ 30 mg/g ein Zielblutdruck von < 130/80 mm Hg vorgeschlagen [111, 112]. Eine Unterstützung für die Wahl dieser Zielwerte ergibt sich aus einer limitierten Anzahl von randomisierten Studien, welche auch Menschen mit Diabetes mellitus beinhalteten und sich auf kardiovaskuläre Ereignisse fokussierten [58]. Allerdings existieren keine randomisierten Studien hinsichtlich Zielblutdruckwerten, die auf renale Ereignisse eingehen. Daten, welche eine Progressionsverzögerung der CKD zeigen, stammen ausschließlich von 3 randomisierten Studien bei Patient*innen ohne DKD, welche Afroamerikaner*innen mit hypertensiver Nephropathie, Patient*innen mit IgA-Nephropathie und Patient*innen mit CKD ohne spezifische Diagnose umfassten [113].

Es gab auch ein Gefahrensignal aus klinischen Studien, dass diastolische Blutdruckwerte < 70 mm Hg und insbesondere < 60 mm Hg bei älteren Menschen problematisch sein könnten [114]. Daten von Patient*innen mit CKD-Stadium G3 oder höher zeigten, dass ein diastolischer Blutdruckwert < 60 mm Hg mit einer erhöhten Inzidenzrate an terminaler Niereninsuffizienz vergesellschaftet ist [115], während andere Studien bei Patient*innen ohne CKD bei diastolischen Werten < 65 mm Hg eine Assoziation mit schlechterem Outcome der kardiovaskulären Erkrankungen zeigten [112, 116].

Ein therapeutischer Nutzen von Blockern des RAAS, sei es durch die Verwendung eines ACE-Hemmers oder Angiotensinrezeptorblockers, ist durch eine Fülle von klinischen Daten nachgewiesen, insbesondere hinsichtlich der Reduktion von renalen Ereignissen bei Patient*innen im CKD-Stadium G3 oder höher, solchen mit einer Albuminurie, Hypertonie und Diabetes mellitus [10, 11, 117]. Daher stellen diese die First-line-Therapie einer antihypertensiven Therapie dar, auch wenn es Hinweise gibt, dass andere Antihypertensiva gleichwertig hinsichtlich harter kardiovaskulärer Endpunkte und dem Auftreten von terminalem Nierenversagen wären [118]. Eine RAAS-Doppelblockade wird nicht empfohlen, da klinische Studien vorzeitig aufgrund von höheren Raten an Hyperkaliämie und/oder akutem Nierenversagen und fehlender Effizienz gestoppt werden mussten [119–121].

## Mineralokortikoidrezeptorantagonisten-Therapie

Nichtsteroidale Mineralokortikoidrezeptorantagonisten (nsMRA) blockieren selektiv den Mineralokortikoidrezeptor, wodurch nachteilige Effekte von Aldosteron wie Entzündung und Fibrose in Herz und Nieren gehemmt werden [122]. Im Gegensatz zu steroidalen MRAs wie Spironolacton wirken sie weniger blutdrucksenkend und bergen ein sehr niedrigeres Risiko für hormonelle Nebenwirkungen (wie z. B. Gynäkomastie, Akne, Libidoverlust etc.). Die FIDELIO-DKD-Studie untersuchte die renalen Effekte von Finerenon, einem nsMRA, bei Personen mit CKD und T2D. Der primäre Endpunkt (Nierenversagen, zumindest 4 Wochen anhaltender Abfall der eGFR um > 40 %, renaler Tod) wurde signifikant um relative 18 % reduziert [123]. Diese renalen Ergebnisse wurden von einer zweiten Interventionsstudie (FIGARO-DKD) mit Finerenon, welche primär kardiovaskuläre Endpunkte untersuchte, bestätigt. Der kombinierte, primäre kardiovaskuläre Endpunkt wurde in dieser Studie um signifikante relative 24 % gesenkt [124]. Das Risiko für akutes Nierenversagen unterschied sich nicht signifikant zwischen der Finerenon- und der Placebogruppe und die Häufigkeit von Hyperkaliämien, die zu einem Studienabbruch führten, lag bei 6 % unter Placebo und 1,7 % in der Finerenon-Gruppe in beiden Studien zusammengefasst [125]. Der maximale Serum-Kalium-Unterschied in der FIDELIO-Kohorte (in welche auch Patient*innen mit höheren CKD-Stadien eingeschlossen wurden) war mit 0,19 mmol/l nach 4 Monaten nur gering [125]. Gepoolte Daten des FIDELIO-DKD- und FIGARO-DKD-Kollektivs (FIDELITY) zeigten ein um 23 % reduziertes Risiko für Endpunkte der CKD-Progression und ein um 22 % reduziertes Risiko einer Hospitalisierung wegen Herzinsuffizienz [125]. Finerenon hatte in FIGARO-DKD und FIDELIO-DKD keinen Einfluss auf HbA_1c_, Körpergewicht oder das Lipidprofil. In der FIDELIO-DKD-Studie führte Finerenon zu einer moderaten Senkung des systolischen Blutdrucks (SBP). Die durchschnittliche Veränderung des SBP betrug etwa −3,84 mm Hg im Vergleich zu Placebo nach 4 Monaten. Die größte Senkung wurde bei Patient*innen mit einem hohen Ausgangs-SBP (> 148 mm Hg) beobachtet mit einem Rückgang von −11,76 mm Hg [123]. In der randomisierten CONFIDENCE-Studie hat eine kombinierte Therapie aus Finerenon und Empagliflozin additiv zu einer bereits etablierten RAAS-Inhibitor-Therapie bei Menschen mit T2D und CKD nach 180 Tagen zu einer stärkeren Reduktion der UACR (29 % bzw. 32 % stärkerer Effekt) verglichen mit einer Monotherapie mit Finerenon bzw. Empagliflozin geführt [126].

Eine Finerenon-Therapie kann bei Menschen mit T2D und CKD G2–4 eingesetzt werden, die trotz einer Therapie mit einem ACE-Hemmer oder Angiotensinrezeptorblocker und einem SGLT-2-Inihibtor für zumindest 4 Wochen eine persistierende Albuminurie (A2–3) und normale Kaliumwerte aufweisen.

Neben den belegten positiven Effekten von Finerenon auf die Nierenfunktion bei Menschen mit T2D [123–125] fehlen derzeit noch Daten bei Menschen mit T1D. In der laufenden FINE-ONE-Studie wird die Wirksamkeit von Finerenon auf das Fortschreiten einer CKD bei Menschen mit T1D untersucht [127]. In der STENO1-Studie wird der Effekt eines multifaktoriellen Managements mit Finerenon, SGLT-1/2-Inhibition oder GLP-1-RA auf renale und kardiovaskuläre Endpunkte bei Menschen mit T1D untersucht (The Steno 1 study. EUCT number: 2023-505794-32).

### Empfehlungen zu Serumkaliumkontrollen unter Finerenon-Therapie

- Bei einer GFR von 25–59 ml/min/1,73 m^2^ sollte mit 10 mg/Tag gestartet werden, bei einer GFR ≥ 60 ml/min/1,73 m^2^ kann mit der Maximaldosis 20 mg/Tag gestartet werden.
- Eine Serumkaliumkontrolle sollte 4 Wochen nach Einleitung und nach weiteren 4 Monaten erfolgen.
- Je nach Serumkalium sollte dann die Therapie mit 10 mg Finerenon pro Tag erhöht oder belassen werden. Bei einem Serumkalium ≥ 5,5 mmol/l sollte Finerenon pausiert werden.

## Zusammenfassung

Zielwerte und Maßnahmen bei DKD:

### Blutdruck

- RR < 140/90 mm Hg
- RR < 130/80 mm Hg bei Albuminurie (Stadium A2 und A3)
- RR diastolisch > 60 mm Hg
- Orthostase vermeiden

### HbA_1c_-Zielwerte

- HbA_1c_-„Zielkorridor“ meistens 6,5–7,5 % (48–58 mmol/mol) (bei fortgeschrittener CKD)
- HbA_1c_-„Zielkorridor“ bei Dialysepatient*innen 7–8,0 % (53–64 mmol/mol). Dieser soll entsprechend dem Alter und der Komorbiditäten individualisiert werden.

### Lipidzielwerte

- Bei Diabetes mellitus mit Albuminurie und/oder ab CKD G3 oder G4/5 ohne Dialyse: LDL-Cholesterin < 55 mg/dl bzw. Non-HDL-Cholesterin < 85 mg/dl bzw. Apolipoprotein B < 65 mg/dl

### Weitere Aspekte

- Hämoglobin 9–11 g/dl (eGFR Stadium CKD G4–5)
- Elektrolyte im Normbereich
- Eiweißzufuhr täglich 0,8 g/kg bis 1,3 g/kg Körpergewicht
- Thrombozytenaggregationshemmer (individuelle Abwägung des potenziellen kardiovaskulären Benefits gegenüber dem Blutungsrisiko)
- Verzicht auf Rauchen
- Exakte Nutzen-Risiko-Abwägung vor Einsatz potenziell nephrotoxischer Medikamente (z. B. nichtsteroidale Antirheumatika, bestimmte Antibiotika wie Aminoglykoside oder Vancomycin)
- Bei Jod-haltigem KM sollte prinzipiell auf eine adäquate Hydrierung geachtet werden, Metformin oder RAASi müssen nicht abgesetzt werden. Neue Gadolinium-haltige KM für MRT-Untersuchungen weisen kein schädigendes Potenzial (nephrogene Sklerose) mehr auf und können daher bedenkenlos bei entsprechender Indikation eingesetzt werden (http://www.esur-cm.org/index.php/en/).
- Beachten der möglichen Kumulation von Begleitmedikamenten
- Beachten des erhöhten kardiovaskulären Risikos mit Screening für Angiopathie
- Beachten von Risiko für Harnwegsinfekte(Restharn?)

### Kontrollen bei Menschen mit DKD

Je nach CKD-Stadium und Progression mindestens 2‑ bis 4‑mal jährliche Kontrollen:

- HbA_1c_, Lipide,
- Bestimmung der Albumin-Kreatinin-Ratio (UACR),
- Bestimmung der Retentionsparameter und Serumelektrolyte (Kreatinin, Harnstoff oder BUN, Kalium),
- Bestimmung der eGFR,
- Blutdruckselbstmessung mit Protokollierung, empfohlen ambulante 24-h-Blutdruckmessung.

Bei einer eGFR < 60 ml/min/1,73 m^2^ zusätzlich (Frequenz vom CKD-Stadium abhängig):

- Blutbild
- Eisenstatus mit Ferritin, Transferrin, Transferrinsättigung, Serumeisen
- Serumphosphat, Serumkalzium, Albumin
- Parathormon, 25-OH-Vitamin D
- Bestimmung der venösen Blutgase insbesondere bei eGFR < 30 ml/min
- Serumkalium (vor allem beim Einsatz von RAS-blockierenden Antihypertensiva und auch Mineralokortikoidrezeptorantagonisten)
- Interdisziplinäre diabetologisch-nephrologische Betreuung ab eGFR < 60 ml/min (Stadium G3) erwägen (Details s. Text oben),
- Hepatitis-B-Virus-Impfschutz
- Bei Auftreten einer akuten Nierenfunktionseinschränkung bzw. Verdacht auf das Vorliegen einer nichtdiabetischen Nierenerkrankung (signifikante Proteinurie) ist eine umgehende nephrologische Begutachtung der Patient*innen zu veranlassen.
- Zur Diagnosesicherung und optimalen Therapieempfehlung ist oftmals eine Nierenbiopsie indiziert. Dieses Vorgehen wird im Einzelfall von der Nephrolog*in mit den Patient*innen besprochen.

## Offenlegungen

H. Sourij hat von folgenden Unternehmen Honorare für Vorträge oder Beratertätigkeit erhalten: Amarin, Amgen, AstraZeneca, Boehringer Ingelheim, Bayer, Daiichi Sankyo, Eli Lilly, Novo Nordisk, Novartis, Sanofi-Aventis, Sobi. F. Aberer hat von folgenden Unternehmen Reiseunterstützung, Honorare für Vorträge, Beratertätigkeit oder Forschungsunterstützungen erhalten: Amgen, Bayer, Sanofi, Novo Nordisk, AstraZeneca und Boehringer Ingelheim. M. Antlanger hat von folgenden Unternehmen Honorare für Vorträge oder Beratertätigkeit erhalten: Boehringer Ingelheim, Bayer, CSL Vifor. J. Brix hat von folgenden Unternehmen Honorare für Vorträge, Beratertätigkeit sowie Forschungsunterstützung erhalten: Abbott, AstraZeneca, Bayer, Boehringer Ingelheim, Dexcom, Eli Lilly, Medtronic, Novo Nordisk. D. Cejka hat von folgenden Unternehmen Honorare für Vorträge erhalten: AstraZeneca, Boehringer Ingelheim und Bayer. M. Clodi hat von folgenden Unternehmen Honorare für Vorträge und Beratertätigkeit erhalten: Novo Nordisk, Elli Lilly, Boehringer Ingelheim, AstraZeneca, Novartis, Daiichi Sankyo, Amgen, Wörwag, Sanofi, Amarin, Roche, Bayer, Sobi. R. Edlinger hat von folgenden Unternehmen Kongressunterstützung, Honorar für Vorträge oder Beratertätigkeit erhalten: Amgen, Astellas, AstraZeneca, Bayer, Boehringer Ingelheim, Fresenius Kabi, Medice, Novartis, Sanofi-Aventis, Stada, Vifor. K. Eller: Beratungs- und Referentenhonorare: CSL Vifor, Böhringer Ingelheim, AstraZeneca, Novo Nordisk, Lilly, Novartis, Alexion, Sanofi, GSK, Chiesi, Otsuka. S. Horn hat von folgenden Unternehmen Honorare für Vorträge oder Beratertätigkeit erhalten: Boehringer Ingelheim, Bayer, CSL Vifor, Otsula, Eli Lilly, Fresenius, AstraZeneca, Sanofi – Aventis, Novo Nordisk. S. Kaser hat Vortragshonorare bzw. Beraterhonorare erhalten von Novo Nordisk, Eli Lilly, Sanofi. A. Kautzky-Willer hat von folgenden Unternehmen, die auch tlw. fördernde Mitglieder der ÖDG sind, Forschungsunterstützungen und/oder Honorare erhalten: Bayer, Boehringer Ingelheim, Amgen, Novartis, Novo Nordisk, Lilly. M. Leutner hat von folgenden Unternehmen, die auch tlw. fördernde Mitglieder der ÖDG sind, Forschungsunterstützungen und/oder Honorare erhalten: Abbott, AstraZeneca, Bayer, Boehringer Ingelheim, Daiichi Sankyo, Novartis, Novo Nordisk. R. Oberbauer hat Vortragshonorare bzw. Beraterhonorare von Neovii, Sanofi, Vifor, Diasorin erhalten. M. Pirklbauer hat von folgenden Unternehmen Reisekostenunterstützung, Honorare für Vorträge oder Beratertätigkeit erhalten: AstraZeneca, Boehringer Ingelheim. D. Strobl hat von folgenden Unternehmen Kongressunterstützung, Honorar für Vorträge oder Beratertätigkeit erhalten: Amgen, AstraZeneca, Bayer, Boehringer Ingelheim, Eli Lilly, Novartis, Novo Nordisk. M. Säemann hat Beratungs- und Sprecherhonorare erhalten von Amgen, AstraZeneca, Bayer, Boehringer Ingelheim, CSL Vifor, Eli Lilly, GSK, Novartis, Novo Nordisk, Otsuka. A.R. Rosenkranz, S. Schmaldienst und H. Stingl geben an, dass kein Interessenkonflikt besteht.
